# Molecular Mechanisms Underlying the Role of MicroRNAs in the Chemoresistance of Pancreatic Cancer

**DOI:** 10.1155/2014/678401

**Published:** 2014-08-28

**Authors:** Ingrid Garajová, Tessa Y. Le Large, Adam E. Frampton, Christian Rolfo, Johannes Voortman, Elisa Giovannetti

**Affiliations:** ^1^Department of Medical Oncology, VU University Medical Center, Cancer Center Amsterdam, De Boelelaan 1117, 1081 HV Amsterdam, The Netherlands; ^2^Department of Experimental, Diagnostic and Speciality Medicine, University of Bologna, Sant'Orsola-Malpighi Hospital, Via Massarenti 9, 40138 Bologna, Italy; ^3^HPB Surgical Unit, Department of Surgery & Cancer, Imperial College, Hammersmith Hospital Campus, White City, London W12 0NN, UK; ^4^Phase I-Early Clinical Trials Unit, Department of Medical Oncology, Antwerp University Hospital, Wilrijkstraat 10, 2650 Edegem, Belgium; ^5^Start-Up Unit, University of Pisa, Lungarno Pacinotti 43, 56126 Pisa, Italy

## Abstract

Pancreatic ductal adenocarcinoma (PDAC) is an extremely severe disease where the mortality and incidence rates are almost identical. This is mainly due to late diagnosis and limited response to current treatments. The tumor macroenvironment/microenvironment have been frequently reported as the major contributors to chemoresistance in PDAC, preventing the drugs from reaching their intended site of action (i.e., the malignant duct cells). However, the recent discovery of microRNAs (miRNAs) has provided new directions for research on mechanisms underlying response to chemotherapy. Due to their tissue-/disease-specific expression and high stability in tissues and biofluids, miRNAs represent new promising diagnostic and prognostic/predictive biomarkers and therapeutic targets. Furthermore, several studies have documented that selected miRNAs, such as miR-21 and miR-34a, may influence response to chemotherapy in several tumor types, including PDAC. In this review, we summarize the current knowledge on the role of miRNAs in PDAC and recent advances in understanding their role in chemoresistance through multiple molecular mechanisms.

## 1. Introduction

A surprising revelation from the human genome project was that 75% of the genome is transcribed into RNA [1–3], but less than 2% is composed of protein-coding genes [4]. Noncoding RNAs (ncRNAs) represent an extremely interesting class of RNAs that can be divided into three types, according to the size. Short ncRNAs are <50 nucleotides (nt); those between 50 nt and 200 nt are referred to as midsize ncRNAs, while long ncRNAs (lncRNAs) are >200 nt [5–8]. miRNAs are a class of short ncRNAs containing approximately 19–24 nt. They have a key regulatory role in development, differentiation, and apoptosis of normal cells, as well as in the determination of the final phenotype of cancer cells, affecting carcinogenesis and metastatic potential [9]. Remarkably, miRNAs exhibit tissue-specific and disease-specific expression that could provide the basis for their development as novel diagnostic, prognostic, and/or predictive biomarkers, as well as therapeutic targets [8]. Furthermore, several studies have documented that selected miRNAs may influence response to chemotherapy [10].

Cancer chemoresistance can occur by multiple mechanisms. It can arise from physiological barriers to drug absorption or penetration into target tissues or from biological mechanisms within individual tumor cells which reduce the effectiveness at their intended site of action, such as increased expression of enzymes involved in drug catabolism or antiapoptotic proteins [10].

The dense stromal reaction which characterizes most PDACs has been frequently reported as the main cause of chemoresistance, preventing the drugs from reaching their intended site of action [11]. However, detailed genetic analyses have unraveled the pivotal mechanisms controlling pancreatic carcinogenesis, and cluster analysis of recurrently mutated genes has defined twelve different core pathways that lead to aberrant signaling in PDAC cells [12]. Such studies suggest that the best hope for the development of agents targeting critical points in the altered pathways lies in the study of mechanisms involved in gene expression regulation. Therefore, in the present review we summarize the role of miRNAs in PDAC and focus on the miRNA-based mechanisms of PDAC chemoresistance.

## 2. Discovery of miRNAs and Their Role in Cancer

The first miRNA molecule, lin-4, was identified in 1993 by Lee and collaborators [13]. In 2000, Reinhart et al. identified lethal-7 (let-7), another miRNA, and discovered its role in the posttranscriptional regulation of gene expression [14]. Currently, it has been reported that there are around 2,600 unique mature human miRNAs (miRBase version 20) [15]. miRNAs regulate more than one-third of all human genes, which suggest their remarkable influence on human biology [16]. It is known that more than 50% of miRNA genes are localized within genomic regions that are either frequently amplified or deleted in different tumor types, resulting in miRNAs deregulation and aberrant expression [17, 18]. The altered miRNAs may have different effects on the tumors [19]. Some of these miRNAs have been characterized as potent oncogenes (oncomiRs), while others have been identified as tumor suppressors (tsmiRs), based on the consequences of their expression on the phenotype of several experimental models [4]. OncomiRs, such as miR-21, are commonly upregulated in cancer [20], while tsmiRs, such as let-7, are downregulated [21], resulting in unique combinations of miRNAs (i.e., overexpressed oncomiRs and underexpressed tsmiRs) characterizing different tumors [22].

The multiple roles of these miRNAs can be explained by starting from the analysis of their biological synthesis and functions. Biosynthesis of miRNAs is a multistep process, involving both nuclear and cytoplasmic components [23]. Initially, they are transcribed in the nucleus by RNA polymerase II into large RNA precursors, called pri-miRNAs [24–26], which can be several hundreds to several thousands of nt in length. The first slicing step performed by the ribonuclease (RNase) III Drosha-DGCR8 (DiGeorge syndrome critical region 8) enzyme leads to the formation of 70-base long pre-miRNAs [27–29]. Pre-miRNAs are actively transported from the nucleus to the cytoplasm by Exportin-5 [30] where they are subjected to further cleavage by the RNase Dicer to achieve the final size, and each molecule is combined with proteins of the Argonaute (AGO) family to obtain its functional form [31–33], thus forming the miRNA-induced silencing complexes (miRISCs). Typically, these complexes bind to the 3′-untranslated region (3′ UTR) of target mRNA with perfect or near-perfect complementarity. When miRNAs form perfect base pairs with their target mRNA, they result in its degradation. Nevertheless, most of the human miRNAs bind to their target 3′-UTRs with imperfect complementarity and therefore induce translational repression [34]. A result of all these interactions is that the target sequence is not translated or there is a variation of translation and subsequently the encoded protein is modified or not produced at all. According to the role of this protein, this leads to structural or functional alterations to the involved cells, thus having a direct effect on their phenotype [9]. Remarkably, each miRNA can regulate the expression of numerous target genes and also the same target gene can be regulated by several types of miRNAs which create a complex network of interactions [35–37]. However, the regulatory role of miRNA in mRNA stability and translation into protein is a complex biological process, which is not restricted through the binding of miRNA only in the 3′-UTR of the mRNA [19]. miRNAs can also interact with the 5′ UTR of protein-coding genes and cause translational repression [38] or activation of the targeted proteins [39]. Similarly, miRNAs can also target the coding sequence and repress the translation of targeted genes [40]. Moreover, some miRNAs can interact with regulatory protein complexes, such as AGO2 and fragile X mental retardation-related protein 1 (FXR1), and indirectly upregulate the translation of a target gene [41].

The involvement of miRNAs in cancer was first discovered in a quest to identify tumor suppressors in the frequently deleted 13q14 region in chronic lymphocytic leukaemia (CLL), and the miRNA cluster miR-15a-miR-16-1 was found to be deleted or downregulated via epigenetic silencing in 69% of the patients [42]. One of the most striking themes in the study of miRNAs and cancer is indeed the large alteration of miRNA expression in malignant cells compared to their normal counterparts. Most cancers have a specific miRNA signature or “miRNome” that characterizes the malignant state and defines some of the clinicopathological features of the tumors (e.g., grade, stage, aggressiveness, vascular invasion, and/or proliferation index) [43]. miRNAs have a variety of roles in cancer development and progression [8], acting not only as tumor suppressors or oncogenes [44], but also as key activators or suppressors of tumor metastasis [45]. Variations in miRNA genes and their precursors, as well as the target sites and genes encoding components of the miRNA processing machinery, can affect the cell phenotype and disease susceptibility [46, 47]. Finally, a subclass of miRNAs, known as epi-miRNAs, can directly control the epigenetic variations [48], and miRNA expression can also be downregulated via promotor hypermethylation [49], adding another piece to the puzzle of regulatory gene expression networks.

Research in various cancers has found that miRNAs also have great potential as biomarkers for early diagnosis and prognosis [17, 50, 51]. In particular, circulating miRNAs have high translational potential as noninvasive biomarkers [52]. Indeed, miRNA expression levels can be detected in a variety of human specimens including both fresh and formalin-fixed paraffin-embedded tissues, fine-needle aspirates, and in almost all human body fluids, including serum, plasma, saliva, urine, and amniotic fluid [53, 54]. The impressive stability of miRNAs in tissues and biofluids is a key advantage over proteins and mRNAs [55]. Circulating miRNAs may have cellular or extracellular origin and are presumably not naked miRNA, which would be degradated within seconds due to the high levels of nucleases in blood. Several reports have demonstrated that stability results from the formation of complexes between circulating miRNAs and specific proteins [56]. Other studies have found miRNAs contained within circulating exosomes or other microvesicles, and it is also possible that cell lysis or an increase in the number of exosomes shed from the diseased cells can contribute to increased levels of certain circulating miRNAs [56]. miRNAs have been found within circulating exosomes or other microvescicles which can be taken up by acceptor cells, playing a role in cell-to-cell communication. Although the mechanism of secretion and incorporation of miRNAs has not been elucidated, secretory miRNAs may play a pivotal role as signaling molecules in physiological and pathological events. In general, there are three mechanisms of shedding which lead to release of vesicles into the extracellular space, that is, via exocytosis, budding of microvesicles directly from a plasma membrane or through the membranous microvesicles shed from cells during apoptosis [56]. However, before applying large-scale efforts to miRNA biomarker discovery, baseline parameters such as intraindividual and interindividual variability of miRNAs must be explored very carefully. Currently there are no validated guidelines for the collection and extraction of samples for miRNA analysis. Differences in specimen types (tissue type or plasma/serum) can have a profound effect on miRNA levels. For example, miRNA content in both plasma and serum can be influenced by cell remnant contamination from erythrocytes, leukocytes, or platelets. Standardization of many analysitical parameters is essential for the evaluation of miRNA as ideal biomarkers.

Further research is also necessary to understand whether miRNAs have clinical potential as prognostic factors and as predictive biomarkers for chemotherapy resistance in specific tumor types. The present review summarizes the current knowledge on the role of miRNAs in PDAC, reporting the most recent studies on miRNA-based mechanisms of chemoresistance.

## 3. miRNA and PDAC

PDAC is a highly aggressive malignancy and fourth leading cause of cancer-related death in developed countries [57]. The median survival after diagnosis is 2–8 months, and approximately only 3–6% of all patients with PDAC survive 5 years after diagnosis [58]. For resectable or borderline resectable patients (i.e., patients with stages T1, T2 or T3 tumors) surgical resection remains the cornerstone of management of PDAC. However, the average survival of resected patients is between 12 and 20 months, with a high probability of relapse [9]. Owing to vague symptoms in early stages, 80% of PDACs are diagnosed when already advanced, and no curative therapy is currently available [59–61].

**Figure 1:**
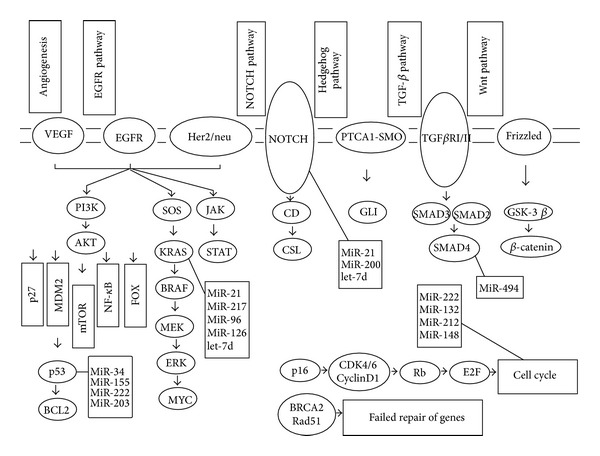
*MicroRNA and their involvement in oncologenic signaling cascades in pancreatic cancer. EGFR pathway.* Activation of the EGF receptor results in autophosphorylation of key tyrosine residues which subsequent activation of downstream signalling cascades including the RAS/extracellular signal regulated kinase (ERK) pathway, the phosphatidylinositol 3-kinase (PI3) pathway and the Janus kinase/Signal transducer and activator of transcription (JAK/STAT) pathway. All of them result in cell survival promotion.* Notch pathway*. A ligand on one cell induces a series of proteolytic cleavage events in a Notch receptor on a contacting cell. These cleavage events release the Notch intracellular domain (NICD), which translocates to the nucleus to activate the transcription of Notch target genes together with CSL (CBF1/Suppressor of Hairless/LAG-1). The notch signaling pathway is important for cell-cell communication, which involves gene regulation mechanisms that control multiple cell differentiation processes.* Hedgehog pathway*. Hedgehog is a secreted ligand that binds to its receptor, Patched (PTCA1). When PTCA1 is activated, it leads to inhibition of the Smoothened (Smo) receptor. Smo is then able to inhibit the phosphorylation and cleavage of Gli, which prevents the formation of repressive Gli (GliR) and promotes the formation of activated Gli (GliA). GliA then translocates into the nucleus and initiates transcription of target genes, which play a role in stem cell regulation.* TGF-*β* pathway*. TGF receptors are activated after binding with their ligand, which leads to further phosphorylation of receptor-regulated SMADs (mainly SMAD2 and SMAD3). Phosphorylated SMAD2/3 form heteromeric complexes with SMAD4, which accumulate in the nucleus and activate transcription of different genes, including those responsible for cell cycle arrest.* Wnt pathway*. In the absence of signal, action of the destruction complex (CKIα, GSK3*β*, APC, and Axin) creates a hyperphosphorylated *β*-catenin, which is a target for ubiqitination and degradation by the proteosome. Binding of Wnt ligand to a Frizzled/LRP-5/6 receptor complex leads to stabilization of hypophosphorylated *β*-catenin, which interacts with TCF/LEF proteins in the nucleus to activate transcription.

Tumors of the pancreas are divided into those arising from the exocrine pancreas and those arising from the endocrine cells. PDACs represent 75% of exocrine malignancies [61]. It has been established that PDAC does not arise* de novo* but is preceded by histologically distinct noninvasive precursor lesions within the pancreatic ducts. The most common precursors are pancreatic intraepithelial neoplasia (PanIN), which show a defined histological progression from the low-grade PanIN-1, through to the intermediate-grade PanIN-2, and culminating in the high-grade PanIN-3 (carcinoma* in situ*) [62]. Key shared genetic alterations associated with PDAC progression include earliest genetic events such as mutation of K-RAS and overexpression of HER-2/neu. At later stages, inactivation of the p16 tumor suppressor gene occurs, followed by the loss of TP53, SMAD4, and BRCA2 signaling pathways and the genomic-transcriptomic alterations that facilitate cell cycle deregulation, cell survival, invasion, and metastases [4]. Importantly, several miRNAs functionally interact with these genetic lesions, as described in the following paragraphs (see also Figure 1).

### 3.1. K-RAS Mutations

Over 90% of PDACs harbor an activating K-RAS gene mutation. The vast majority of these mutations are at codon 12 and occur very early in pancreatic carcinogenesis [63]. K-RAS is a 21 kDa intracellular membrane bound protein that belongs to the GTPase superfamily [64, 65]. In physiological conditions, the GAP proteins and, specifically, the RAS GTPases do promote GTP hydrolysis and reversal of the RAS activation step [66]. During oncogenic transformation, the mutated RAS is constitutively activated and cannot be deactivated by the GAP proteins [65]. RAS signaling involves multiple branches (B-RAF, PI3K, and PLC pathways). Together, these branches cover most aspects of cellular life, including regulation of the cell cycle, differentiation, proliferation, and apoptosis [62]. Several recent studies have identified specific miRNAs that regulate the K-RAS signaling pathway in pancreatic oncogenesis and vice versa. Preclinical studies have shown that K-RAS regulates miR-21 expression levels in precancerous lesions and the peak of miR-21 expression correlates with the degree of progression to more aggressive forms [67]. K-RAS is also a direct target of miR-217; thus upregulation of miR-217 decreases K-RAS protein levels and reduces the constitutive phosphorylation of downstream AKT [68]. Another study identified K-RAS as a direct target of miR-96 [69]. Indeed, overexpression of miR-96 decreased cancer cell invasion, migration and slowed tumor growth and was associated with K-RAS downregulation [69]. Recent studies have shown that miR-126 and let-7d can also regulate K-RAS levels in PDAC. In particular miR-126 can directly target K-RAS; thus miR-126 downregulation can allow overexpression of K-RAS [70].

### 3.2. HER2/neu Overexpression

Up to 29% of PDACs have HER2 overexpression [71–73]. There is direct correlation between the expression levels of the Her2/neu and the shorter survival in patients with PDAC, suggesting that the HER2/neu signaling pathway is a central regulator of pancreatic oncogenesis [74].

The HER2/neu pathway has been primarily studied in breast cancer cell lines, where miR-21 expression levels correlate with the HER2/neu upregulation [65]. More recently, dysregulation of miR-125a-5p/125b and HER2 emerged as an early event in the gastric (intestinal-type) and esophageal (Barretts) oncogenesis [75]. In these oncogenic lesions, miR-125 expression correlates inversely with HER2 status. Therefore, miR-125a-5p/125b can be considered among the therapeutic targets in HER2-positive esophageal and gastric adenocarcinoma. Similarly, the role of newer anti-HER2 agents agents interacting with regulating miRNA in HER2-positive PDAC remains to be explored [74, 76].

### 3.3. p16/CDKN2A Inactivation

CDKN2A is a tumor suppressor gene which is somatically inactivated in approximately 95% of PDACs [77]. Most of these inactivating mutations lead to loss of function of the protein p16, the product of the CDKN2A gene. The p16 protein binds cyclin-dependent kinases 4 and 6 (CDK4 and CDK6) and specifically inhibits their pRb phosphorylating activity, which is required for G1/S transition [62]. Inherited mutations in the p16/CDKN2A gene cause the familial atypical multiple mole melanoma syndrome, with increased risk for developing PDAC and melanoma [78]. Several miRNAs that participate in the deregulation of the cell cycle genes are essential during PDAC development and progression. For example, miR-222 targets p27 and p57, which are both pivotal cell cycle inhibitors [79]. Other studies have shown that downregulation of miR-132 and miR-212 causes G2/M cell cycle arrest and results in reduced cell proliferation [80], while miR-148 directly targets AMP activated protein kinase (AMPK), which plays a key role as a master regulator of cellular energy homeostasis, and can induce cell cycle arrest and apoptosis [81].

### 3.4. TP53 Mutations

The TP53 gene is inactivated in 75 to 85% of PDACs [63]. Genetic inactivation of TP53 abrogates important cell functions, such as regulation of cellular proliferation and apoptosis in response to DNA damage. When cellular stress and DNA damage are detected, degradation of TP53 is inhibited by different mechanisms, leading to accumulation of its active form [82]. Preclinical studies have shown that TP53 directly regulates miR-34, which further downstream targets Notch, and therefore plays a role in the maintenance and survival of PDAC initiating cells [82]. Moreover, TP53-induced nuclear protein 1 gene has been described to be downregulated by miR-155, accelerating pancreatic tumor development [83]. MiR-222 and miR-203 are also able to target p53 and affect its function as a crucial regulator of the cell cycle [84].

### 3.5. SMAD4 Inactivation

The SMAD4 gene is inactivated in approximately 60% of PDACs [63]. The protein product of the SMAD4 gene is involved in the transmission of intracellular signals from transforming growth factor beta (TGFb) receptors within the cell membrane to the nucleus [85]. In normal cells, TGF-A receptors are activated after binding with their ligand, which leads to further phosphorylation of receptor-regulated SMADs (mainly SMAD2 and SMAD3). Phosphorylated SMAD2 and SMAD3 form heteromeric complexes with SMAD4, which accumulate in the nucleus and activate transcription of different genes, including those responsible for cell cycle arrest. This pathway is of key importance for pancreatic cells [62]. PDACs with loss of SMAD4 expression have higher rates of distant metastases and a poorer prognosis [86, 87]. A recent study showed that loss of SMAD4 in PDAC cells leads to increased levels of FOXM1, nuclear localization of *β*-catenin, and reduced levels of miR-494 [88]. Transgenic expression of miR-494 in PDAC cells produced the same effects as reducing expression of FOXM1 or blocking nuclear translocation of *β*-catenin, reducing cell proliferation, migration, and invasion, and increasing their sensitivity to gemcitabine. Reduced expression of miR-494 correlated with PDAC metastasis and reduced survival times of patients. This study suggested that miR-494 might be developed as a prognostic marker or a therapeutic target for patients with PDAC. Other studies have shown that in human PDAC specimens, the expression levels of both miR-421 and miR-483-3p are inversely correlated to SMAD4 expression and ectopic expression of these miRNAs significantly represses SMAD4 protein levels in PDAC cell lines, suggesting that they are potent regulators of SMAD4 in PDAC [89, 90].

### 3.6. BRCA2 and PALB2 Mutations

The BRCA2 gene is inactivated in fewer than 10% of PDACs [91]. Importantly, germline mutations in BRCA2 are associated with an increased risk of PDAC [92]. Similarly, germline truncating mutations in the PALB2 gene, which encodes for a BRCA2 binding protein [93], have been identified in ~3% of individuals with familial pancreatic cancer [94, 95]. Of note, a recent study for the prediction of BRCA1/2 mutation-associated hereditary breast cancer identified a 35-miRNA classifier for the prediction of BRCA1/2 mutation status with a reported 95% and 92% accuracy in the training and the test set, respectively [96]. These miRNA signatures might be of interest also in PDAC, in order to complement current patient selection criteria for gene testing by identifying individuals with high likelihood of being BRCA1/2 mutation carriers.

## 4. MicroRNA-Based Mechanisms of Anticancer Drug Resistance in PDAC

Chemotherapy remains the primary treatment for metastatic, nonresecable PDAC. However, the best currently available treatments prolong life by only a few months [97, 98], and PDAC chemoresistance renders most drugs ineffective.

Drug resistance can be divided into two groups: intrinsic or acquired. Intrinsic resistance is caused by a preexisting phenotype, whereas acquired resistance develops due to repeated use of the same drug. The most common reason for the acquisition of resistance to a broad range of anticancer drugs is the overexpression of one or more energy-dependent transporters that detect and eject anticancer drugs from cells, resulting in multidrug resistance (MDR) [10, 99]. However, drug resistance can occur for many causes, including increased drug efflux, alterations in drug target, DNA repair, cell cycle regulation, and evasion of apoptosis [100].

**Figure 2:**
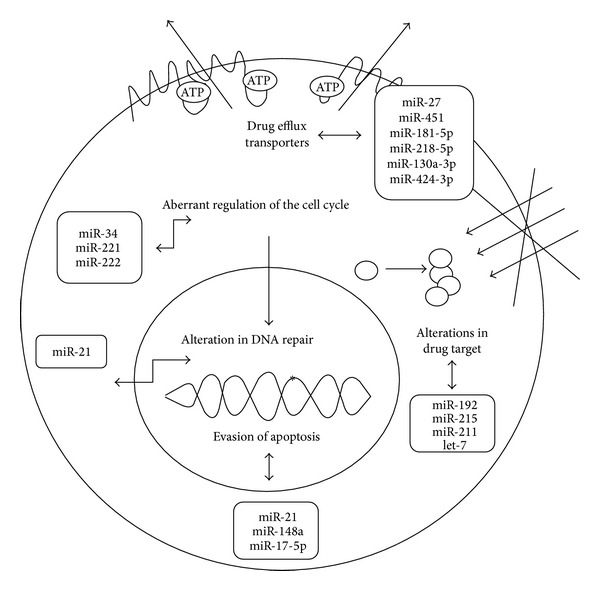
*MicroRNA and their involvement in anticancer drug resistance*. Drug resistance can occur at many levels, including drug efflux, alterations in drug target, DNA repair, cell cycle regulation, and evasion of apoptosis. Some selected miRNAs which have been demonstrated to alter these mechanisms are shown in this figure.

Up- and/or downregulation of miRNAs can influence the expression of multiple target mRNAs, and therefore multiple proteins, leading to variations in the chemosensitivity of cancer cells via various cellular processes. In particular, several miRNAs have been demonstrated to alter cellular response to anticancer agents via modulation of drug efflux and targets, cell cycle, survival pathways, and/or apoptotic response, as reported in the following paragraphs and in Figure 2.

### 4.1. Upregulation of Drug Efflux Transporters

Resistance to various anticancer agents has been associated with increased expression of drug efflux pumps [99], keeping the intracellular drug concentration below a cell-killing threshold [100]. miRNAs have also been shown to be involved in chemotherapy resistance through the regulation of ATP-binding cassette (ABC) membrane transporters [100]. They transport drugs from the cytosol to the extracellular space. Activation of the MDR1 gene results in overexpression of P-glycoprotein (P-gp) which is a multidrug efflux pump and confers cancer cell resistance to a broad spectrum of drugs [10, 101]. P-glycoprotein is localized at the apical level in cells membranes of different cellular compartments such as liver, intestine, kidney, and placenta. This strategic localization gives P-gp a crucial role as responsible for drugs absorption and accumulation [101]. It has been shown that miR-27a and miR-451 are activators of drug resistant process by modulation of MDR1/P-gp expression in human ovarian and cervical cancer cells [10, 102]. A recent study evaluated the role of miRNAs in MDR in PDAC, monitoring the modulation of some specific miRNAs by the treatment of a wild type cell line and in the corresponding cell line with P-gp overexpression and unsensitive to several antineoplastic treatments [103]. This study showed the different modulation of 4 miRNAs (miR-181a-5p, miR-218-5p, miR-130a-3p, and miR-424-3p), using a specific P-gp substrate, and suggested new molecular mechanisms potentially involved in chemoresistance, such as the modulation by miR-424 of the protein cullin 2, a scaffolding protein displaying a pivotal role in the assembly of the ubiquitin ligase system, thereby stabilizing HIF-1α.

### 4.2. Alterations in Drug Targets and DNA Repair

Chemoresistance can be caused by either quantitative (i.e., modulation of expression levels) or qualitative (i.e., mutation) alterations of the drug targets [100]. Examples of quantitative alterations have been reported for several antimetabolites, which influence various steps the metabolism of nucleic acids, through inhibition of key enzymes, such as thymidylate synthase and ribonucleotide reductase. MiR-192 and miR-215 target thymidylate synthase (TS), which is the main drug target of the fluoropyrimidine-based therapy in colorectal cancer, which is also used in PDAC patients [104]. However, downregulation of TS by miR-192/215 did not lead to an increase in 5-FU sensitivity, suggesting that the activity of miR-192/215 was not mediated by TS. In contrast, overexpression of both miRNAs resulted in a reduction of cell proliferation and therefore diminished the effectiveness of S-phase specific drugs like 5-FU, suggesting that miR-192 and miR-215 can still play a role in 5-FU resistance.

Two recent studies suggested the key role of miR-211 in the modulation of ribonucleotide reductase subunit 2 (RRM2), which is an important cellular target of gemcitabine. This miRNA had significantly higher expression in long- versus short-OS PDAC patients, evaluating high-resolution miRNA profiles with Toray's 3D-Gene-miRNA-chip, detecting more than 1200 human miRNAs [105]. The preclinical analyses demonstrated that induction of the miR-211 expression in PDAC cells increased the sensitivity to gemcitabine through reduced expression of its target RRM2 [106]. Similarly, it has been demonstrated that let-7 negatively regulates RRM2 and let-7 expression is inversely correlated with RRM2 expression in gemcitabine-resistant PDAC cells. Additionally, silencing RRM2 or overexpression of let-7 was shown to sensitize PDAC cells to gemcitabine [107].

miRNAs can also alter cellular response to several anticancer drugs via interfering with DNA repair. In particular, the inhibition of ribonucleotide reductase by gemcitabine results in deoxyadenosine triphosphate depletion, causing DNA replication errors. Moreover, gemcitabine is incorporated into DNA and arrests DNA replication. Both the mispaired bases and the gemcitabine-modified DNA bases can be the substrates for postreplicative DNA mismatch repair (MMR) machinery [108], which influences cancer cell sensitivity.

Similarly, defects in MMR proteins have been associated with reduced or absent benefit from 5-FU adjuvant chemotherapy [109]. MMR alterations reduce the incorporation into DNA of the 5-FU metabolites that cause G2/M arrest and induce apoptosis after 5-FU treatment. Colorectal cancer cells with miR-21 overexpression exhibited significantly reduced 5-FU-induced G2/M damage arrest and apoptosis, suggesting that miR-21-dependent downregulation of core MMR component (hMSH2–hMSH6) might be responsible for both primary and acquired resistance to 5-FU [110, 111]. Of note, miR-21 is included in the miRNA metasignature for recognising PDAC [112, 113]. Furthermore, high miR-21, high miR-31, and low miR-375 tumoral expressions have been validated as independent prognostic biomarkers for poor overall survival in PDAC.

### 4.3. Aberrant Regulation of the Cell Cycle

The cell cycle is an ordered set of events, culminating in cell growth and division into two daughter cells. Uncontrolled cellular proliferation is one of the hallmarks of cancer, and these alterations are commonly caused by genetic damages to regulator genes such p16 and cyclin D1 that govern phosphorylation of the retinoblastoma protein (RB) and control exit from the G1 phase of the cell cycle or the tumor suppressor TP53, which can arrest growth by holding the cell cycle at the G1/S regulation point on DNA damage recognition [114]. Recent studies showed that the members of the miR-34 family are direct TP53 targets, and their upregulation induced apoptosis and cell cycle arrest [115]. The miR-34 family comprises three miRNAs, encoded by two different genes: miR-34a is encoded by its own transcript, whereas miR-34b miR-34c share a common primary transcript. Moreover, the promoter region of miR-34a, miR -34b, and miR -34c contains CpG islands. An aberrant CpG methylation reduces miR-34 family expression in multiple types of cancer, including PDAC [116]. Therefore a recent study investigated the functional significance of miR-34a in PDAC progression through its epigenetic restoration with chromatin modulators, demethylating agent 5-Aza-2′-deoxycytidine, and HDAC inhibitor Vorinostat [117]. The restoration of miR-34a in human PDAC and pancreatic cancer stem cells (CSCs) strongly inhibited cell proliferation, cell cycle progression, self-renewal, epithelial to mesenchymal transition, and invasion, while inducing apoptosis. These results provided not only mechanistic insight but also promising therapeutic approaches, which might also improve esponse to existing chemotherapies in PDAC.

Another example of protein of interaction between proteins regulating the cell cycle and miRNA is represented by Cyclin-dependent kinase inhibitor 1B (CDKN1B, p27, or p27Kip1), which is a cell cycle inhibitor and tumor suppressor. This enzyme has been identified as a direct target of miR-221 and miR-222 [53]. The expression of miR-221 is significantly upregulated in PDAC cell lines and tumor tissues compared to normal pancreatic duct epithelial cells and normal pancreas tissues and has been proposed as candidate plasma biomarkers in PDAC [118]. However, transfection of miR-221 inhibitor suppressed the proliferative capacity of PDAC cells with concomitant upregulation of CDKN1B, as well as of PTEN and PUMA, which are other tumor suppressors among the predicted targets of miR-221 [119]. The same study showed that the expression of miR-221 was modulated by the treatment with isoflavone mixture (G2535), formulated 3,3′-diindolylmethane (BR-DIM), or synthetic curcumin analogue (CDF), leading to the inhibition of cell proliferation and migration and supporting further studies on these potential nontoxic agents in novel targeted therapeutic strategy that are capable of downregulation of miR-221.

### 4.4. Evasion of Apoptosis

Apoptotic evasion is considered to be one of the main causes of chemotherapeutic and radiotherapeutic resistance that characterizes the most aggressive tumor [120]. Cancer cells can resist apoptosis if they have an overexpression of antiapoptotic proteins, involved in the two main apoptosis pathways, extrinsic and intrinsic. The extrinsic pathway is regulated mainly by “death receptors” of the TNF-receptor family, while the intrinsic pathway is regulated by Bcl-2 proteins. Various anticancer drugs such as antimetabolites, DNA cross-linking and intercalating agents, alkylating agents, topoisomerase I/II inhibitors, and TKIs have been reported to induce intrinsic or extrinsic apoptotic response in tumor cells, resulting in caspases activation [121]. Although the extrinsic and the intrinsic apoptosis pathways are activated by different stimuli, both these pathways can be regulated by specific miRNAs. For example, upregulation of Bcl-2, directly induced by miR-21, is associated with apoptosis, chemoresistance to gemcitabine, and proliferation of MIA PaCa-2 cells [110]. Using western blot and luciferase activity assay, Bcl-2 was identified also as a target of miR-148a, and the expression of Bcl-2 lacking in 3′UTR could abrogate the proapoptotic function of miR-148a in PANC-1 and AsPC-1 cells [122]. Similarly, exogenous expression of miR-204 and miR-320 reduced the protein level of their targets, Bcl-2 and Mcl-1, respectively. Mcl-1 is an antiapoptotic member of Bcl-2 family, and induction of miR-320 activity leads to apoptosis through Mcl-1 suppression, sensitizing cholangiocarcinoma cells to 5-FU [123]. However, miR-204 was also reported to be significantly downregulated in gemcitabine-resistant PDAC [124], and Li et al. identified the role of the entire miR-200 family of miRNAs in gemcitabine-resistant PDAC cells [125].

Conversely, miR-17-5p downregulates the proapoptotic member of the Bcl-2 protein family Bim, and PDAC cells transfected with miR-17-5p inhibitor showed growth inhibition, spontaneous apoptosis, higher caspase-3 activation, and increased chemosensitivity to gemcitabine [126]. Pathways delivering an antiapoptotic signal, such as PI3K/Akt, play also a pivotal role in the balance between proapoptotic and survival signals, which determines the fate of cancer cells. An increased miR-21 expression has been associated with the activation of PI3K/AKT/mTOR pathway, while combination of anti-miR-21 strategies with drugs targeting PI3K/AKT/mTOR pathway reduced pAKT levels and enhanced apoptosis when used in combination with gemcitabine [127]. Importantly, the antiapoptotic role of miR-21 is possibly tumor specific, with inhibition of miR-21 increasing sensitivity and apoptosis induction by gemcitabine in PDAC and cholangiocarcinoma, but not in colon cancer cells [127]. This suggests that its oncogenic properties could be cell and tissue dependent and that its potential role in chemoresistance should be contextualized with respect to the tumor type and the treatment [128].

## 5. miRNA in PDAC Resistance to Conventional Therapy and Target Therapy

Pancreatic cancer is a genetically heterogenous disease with a very limited response to most treatments [129], including both conventional (also known as standard-dose chemotherapy, which includes chemotherapeutic agents and regimens that have been in use from the past 15 to 40 years) and targeted therapies (a newer type of cancer treatment that uses drugs or other substances to more precisely identify specific molecules involved in cell growth and survival and attack cancer cells) as described in the following paragraphs.

### 5.1. Conventional Chemotherapy

Conventional chemotherapy, also known as standard-dose chemotherapy, includes chemotherapeutic agents and regimens that have been in use from the past 15 to 40 years. The three different therapeutic options for PDAC in the metastatic setting include gemcitabine, as monotherapy or in combinations: the combination of 5-FU, leucovorin, irinotecan, and oxaliplatin (FOLFIRINOX), and the most recent combination of gemcitabine with nab-paclitaxel. Although only 20% of patients present with localized disease amenable to potentially curative resection, on the basis of a few randomized trials [130–132], the current accepted standard of care is adjuvant gemcitabine or 5-FU chemotherapy, while there have been no conclusions regarding the role or timing of adjuvant chemoradiation [133].

### 5.2. Gemcitabine Monotherapy and Gemcitabine-Based Combinations

Since 1997, gemcitabine is being used in metastatic PDAC. Patients receiving gemcitabine have a median survival of 6.2 months and a 1-year survival rate of 20% [134]. Meta-analysis of randomized trials with a combination of gemcitabine and platinum analogues or of gemcitabine and capecitabine suggested a survival benefit for these combinations for patients with a good performance status [135–137]. In contrast, an Italian phase III trial examining gemcitabine and cisplatin did not confirm a survival benefit for this combination [138]. In a retrospective study on laser-microdissected PDAC specimens patients with high miR-21 expression had a significantly shorter overall survival both in the metastatic and in the adjuvant setting. Multivariate analysis confirmed the prognostic significance of miR-21 [127]. The reduced expression of miR-21 was associated with benefit from gemcitabine treatment in two independent cohorts of PDAC patients [139, 140], as well as in a cohort of intraductal papillary mucinous neoplasms (IPMNs) of the pancreas [141]. These results might be explained by the effects of miR-21 expression on certain phenotypic characteristics in PDAC cell lines [139, 142]. Overexpression of miR-21 promotes cell proliferation, increases the metastatic ability through expression of matrix metalloproteinase-2 and metalloproteinase-9 as well as VEGF, and decreases gemcitabine sensitivity, whereas miR-21 repression delivers the opposite results [143]. Furthermore, as reported in the previous chapters, Hwang et al. [139] and Dong et al. [144] provided experimental evidence for a role of miR-21 in chemoresistance thorough modulation of apoptosis by directly regulating Bcl-2 and PTEN expression. More recently, Frampton et al. identified three miRNAs (miR-21, miR-23a, and miR-27a) that acted as cooperative repressors of a network of tumor suppressor genes that included PDCD4, BTG2, and NEDD4L [145]. In 91 PDAC samples from PDAC radically resected patients, high levels of a combination of these miRNAs were associated with shorter survival times. Thus, high expressors of this triple miRNA combination (miR-21/23a/27a) may be identified as having a much worse prognosis and may benefit from anti-miRNA therapy, although the best way to deliver such a treatment and potential off-target effects are unknown. Another recent study demonstrated that miR-10b might be a novel diagnostic and predictive biomarker for PDAC [146]. MiR-10b is indeed overexpressed in PDAC patients and reduced expression of miR-10b was associated with improved response to multimodality neoadjuvant therapy, likelihood of surgical resection, delayed time to metastasis, and increased survival [146]. Finally, several studies reported miR-155, among the miRNA which are commonly overexpressed in PDACs and their precursor lesions [147], and although only one study reported that its elevated expression correlated with shorter survival [84], Xia et al. [148] demonstrated that gemcitabine treatment induced the expression of miR-155 in PDAC cells suggesting its role in acquired chemoresistance. Other miRNAs that have been linked to gemcitabine chemoresistance in PDAC are reported in Table 1.

Gemcitabine plus nanoparticle albumin-bound nab-paclitaxel represents a novel, acceptable alternative to FOLFIRINOX. This combined therapy was associated with significantly higher objective response rate (23%) and significantly longer median overall (8.5 months) and progression-free survival (5.5 months), in comparison to gemcitabine alone [149]. Combination treatment with gemcitabine and nab-paclitaxel increases intratumoral gemcitabine levels attributable to a marked decrease in the primary gemcitabine metabolizing enzyme, cytidine deaminase. Correspondingly, paclitaxel reduced the levels of cytidine deaminase protein in cultured cells through reactive oxygen species-mediated degradation, resulting in the increased stabilization of gemcitabine [150]. Nab-paclitaxel alone or in combination with gemcitabine has been demostrated to reduce the desmoplastic stroma [151]. Moreover, it is hypothesized that the albumin-bound nab-paclitaxel may selectively accumulate in the pancreatic stroma via its binding to secreted protein acidic and rich in cysteine (SPARC) matricellular glycoprotein which binds albumin and is overexpressed in tumor stroma [57]. High SPARC expression has been correlated to poor survival outcome and has been suggested as a possible predictive biomarker for nab-paclitaxel in the phase-II trial [151]. However, no data on SPARC are available from the phase III trial and Neesse et al. showed that the effects of nab-paclitaxel were largely dose-dependent and that SPARC expression in the tumor stroma did not influence drug accumulation in a PDAC mouse model. Further studies are therefore warranted to evaluate tissue and plasma SPARC expression as a potential predictive biomarker for nab-paclitaxel [11].

No data are available on miRNA affecting nab-paclitaxel, but several miRNAs have been associated to resistance to paclitaxel. Regarding miRNA potentially affecting the drug target, TUBB3 has been unraveled as a target for miR-200c in ovarian and endometrial cancer cells, and the ectopic expression of this miRNA downregulated TUBB3 and enhanced sensitivity to microtubule-targeting agents, including paclitaxel [152].

As example of miRNA affecting survival pathway, miR-17-5p has been identified as one of most significantly downregulated miRNAs in paclitaxel-resistant lung cancer cells, which might cause upregulation of beclin 1 gene, one of the most important autophagy modulators [153]. Moreover, miRNA miR-17-5p, which is a member of the miR-17-92 cluster, is upregulated in pancreatic cancer and some present findings suggest that miR-17-5p plays important roles in pancreatic carcinogenesis and cancer progression and is associated with a poor prognosis in pancreatic cancer [154].

### 5.3. FOLFIRINOX (5-FU, Leucovorin, Irinotecan, and Oxaliplatin)

A phase III trial using FOLFIRINOX regimen in PDAC patients has shown a response rate of 31.6%, a median survival of 11.1 months [155]. Therefore, FOLFIRINOX protocol confers a significant improvement in the overall survival in stage IV PDAC and can be considered as a novel therapeutic option for patients with a good performance status [136]. No predictive biomarkers are actually used in clinical practice, but a few studies suggested the role of candidate miRNAs to predict the sensitivity/resistance to 5-FU, and the other drugs in this regimen. 5-FU activity might indeed depend on the expression of its target TS, or by the modulation of cell cycle, and apoptosis induction by several miRNAs, as reported above.

Interestingly, a pharmacogenetic study evaluated 18 polymorphisms both in miRNA-containing genomic regions (primary and precursor miRNA) and in genes related to miRNA biogenesis with outcome in metastatic colorectal cancer patients treated with 5-FU and irinotecan [156]. A significant association with tumor response and time to progression was observed for the SNP rs7372209 in pri-miR26a-1. The genotypes CC and CT were favorable when compared with the TT variant genotype. Similarly, the SNP rs1834306, located in the pri-miR-100 gene, significantly correlated with a longer time to progression.

### 5.4. Targeted Therapy

From its introduction, cancer chemotherapy has been encumbered by its poor selectivity because most antineoplastic drugs are toxic also to fast-replicating cells of the blood compartment, skin cells, and gastrointestinal tract lining cells. This unsatisfactory situation and the development of technology leading to the sequencing of the genome have driven intensive researches and development over the last few decades towards more specific and less toxic anticancer drugs that block the growth and spread of cancer by interfering with specific molecules involved in tumor growth and progression and are therefore called “targeted therapies.” Some of these therapeutic regimens especially designed to intercept deregulated dominant oncogenes have proven to be effective treatment in “oncogene addicted” tumors [157]. In particular, the epidermal growth factor receptor (EGFR) has been successfully targeted either by mAbs or small molecules inhibiting the tyrosine kinase domain. The mAb cetuximab blocks the extracellular domain of EGFR, thereby competing with the ligands and resulting in the inhibition of the receptor. This mAb is approved for the treatment of advanced colorectal cancer, while the EGFR-TKIs gefitinib and erlotinib have been approved as upfront therapy replacing chemotherapy in late-stage NSCLC patients harboring activating-EGFR mutations.

### 5.5. Anti-EGFR Therapy in PDAC

The SWOG group conducted a randomized Phase III clinical trial randomizing patients with stages III-IV PDAC to receive either gemcitabine alone or in combination with cetuximab, which did not improve the clinical outcome. Negative results for this combination were also observed in the adjuvant setting [158]. Similarly, other EGFR and HER2 targeted therapies, including trastuzumab and lapatinib, have not shown a survival benefit in PDAC patients [136]. In contrast, a combination of gemcitabine and erlotinib has been approved for use by the United States Food and Drug Administration (FDA) and European Medicines Agency (EMEA) as a treatment for PDAC patients on the basis of a randomized trial, showing a overall gain in median survival of 2 weeks [159]. Examination of K-RAS mutational status and EGFR gene copy number in 26% of patients from this trial failed to identify either change as molecular predictors of response [160]. However, accumulating evidence suggests that dysregulation of specific miRNAs may be involved in the acquisition of cancer cell resistance to EGFR-targeted agents. In particular, miR-7 emerged as a critical modulator of a regulatory network for EGFR signaling in lung cancer cells, with the ability of coordinately downregulating the expression of several members of the EGFR signaling cascade [161]. The binding of c-Myc to the miR-7 promoter enhanced its activity, while ectopic miR-7 promoted cell growth and orthotopic tumor formation in nude mice. In these models, quantitative proteomic analysis revealed that miR-7 decreased levels of the Ets2 transcriptional repression factor ERF, which is a direct target of miR-7. Accordingly, the inhibition of miR-7 expression suppressed EGFR mRNA and protein expression in different lung cancer cell lines as well as the growth of the A549 lung adenocarcinoma cells [162]. Of note, miR-7 is preferentially expressed in endocrine cells of the developing and adult human pancreas [163]. However, its role in the regulation of the insulin growth factor-1 receptor expression might affect the development of diabetes-associated PDAC [164].

Other studies in lung cancer cell lines showed that decreased miR-424 levels were indicative of increased resistance to erlotinib, while the gefitinib resistant cell line-HCC827/GR had a significant upregulation of miR-214 [165]. The inhibition of miR-214 has been also correlated with decreased apoptosis and miR-214 and PTEN were indeed inversely expressed, while knockdown of miR-214 altered the expression of PTEN and p-AKT, resensitizing HCC827/GR to gefitinib. MiR-214 has been identified as aberrantly expressed in PDAC and* in vitro* experiments showed that overexpression of miR-214 decreased the sensitivity of the BxCP-3 cells to gemcitabine [166].

The sensitivity to erlotinib was also predicted by a 13-gene miRNA signature, identified in sensitive towards resistant lung cancer cell lines. Ontological annotation of these miRNA (miR-140-3p, miR-628-5p, miR-518f, miR-636, miR-301a, miR-34c, miR-224, miR-197, miR-205, miR135b, miR-200b, miR-200c, and miR-141) and their potential targets revealed enrichment in the components of EMT, including Wnt pathway, which may explain the ability of this signature to separate primary from metastatic tumor samples as well as why the treatment with TGF*β*1 modulated both the expression of these miRNA and cell migration [167]. Interestingly, EMT has been inversely correlated with the response of cancers to EGFR-targeted therapy and the TGF*β*-mitogen-inducible gene 6-miR200 network orchestrates the EMT-associated kinase switch hat induces resistance to EGFR inhibitors in primary tumor xenografts of patient-derived lung and pancreatic cancers carrying wild type EGFR [168]. These data support the low ratio of Mig6 to miR200 as a promising predictive biomarker of the response of PDAC to EGFR-TKIs.

## 6. miRNA Affecting PDAC Chemoresistance through Modulation of Its Microenvironment

PDAC is characterized by a dense fibrotic stromal matrix [11], composed of activated fibroblasts/stellate cells, inflammatory cells, and other cell types such as endothelial cells. PDAC is one of the most stroma-rich malignancies [169]. Such desmoplasia facilitates a mechanopathology known as growth-induced solid stress, resulting in collapsed or compressed intratumoral blood vessels or lymphatics, which respectively lead to increased hypoxia and interstitial fluid pressure, both attenuating chemosensitivity [170].

Hypoxia is an essential component of the PDAC microenvironment, as demonstrated by the characteristic avascular appearance on computed tomography and low oxygen tension measurements of these tumors [171, 172]. Several studies showed that hypoxia plays a pivotal role in cancer progression through induction of the hypoxia-inducible factor (HIF), which leads to increased expression of VEGF [173]. However, hypoxic conditions in solid malignancies may also confer resistance to conventional radiation and chemotherapy [174]. A functional link between hypoxia and miRNA expression was shown in colon and breast cancer cell lines [175] and in several other cancers, including PDAC [176]. MiR-210, in particular, is induced by hypoxia and the levels of this miRNA are significantly higher elevated in PDAC patients and may potentially serve as a useful biomarker for PDAC diagnosis [177]. Furthermore, miR-210 regulates the interaction between PDAC cells and stellate cells, promoting the progression and chemoresistance of tumor cells [178]. However, the same study showed that stellate cells-induced miR-210 upregulation was inhibited by inhibitors of ERK and PI3K/Akt pathways, suggesting novel therapeutic combinations to counteract the interaction between stellate cells and PDAC, which is at least in part responsible for the innate resistance to chemotherapy in pancreatic tumors by creating barriers against circulating therapeutic compounds.

Hypoxia induces also the overexpression of miR-21 [179], while the treatment with the novel curcumin-derived analogue CDF downregulated the expression of miR-21 and miR-210, as well as Nanog, Oct4, and EZH2 mRNAs, and the production of VEGF and IL-6. CDF also led to decreased cell migration/invasion, angiogenesis, and formation of pancreatospheres under hypoxia, supporting further studies on its role to overcome microenvironment-mediated chemoresistance of PDAC [180].

Other important factors playing a key role in PDAC microenvironment and chemoresistance include cells of the immune response and CSCs. Recent data indicated that tumor-associated macrophages (TAMs), which are abundant in the microenvironment of PDAC, secrete protumorigenic factors that contribute not only to cancer progression and dissemination but also to chemoresistance by reducing gemcitabine-induced apoptosis. In particular, TAMs induce upregulation of cytidine deaminase, the enzyme that metabolizes gemcitabine following its transport into the cell [181]. Moreover, immune cells within the tumor microenvironment can also activate pancreatic stellate cells which orchestrate the strong desmoplasia that characterizes PDAC and the resulting hypoxia [182]. Importantly, several miRNAs, including miR-155, which is commonly overexpressed in PDAC, are involved in the control of macrophage production and activation, suggesting that reprogramming miRNA activity in TAMs and/or their precursors might be effective for controlling tumor progression/chemosensitivity [183].

The existence of CSCs has been widely accepted to be responsible for tumor aggressiveness in PDAC, because CSCs have the capacity for increased cell growth, cell migration/invasion, metastasis, and also treatment resistance. However, a recent study detected deregulated expression of over 400 miRNAs, including let-7, miR-30, miR-125b, and miR-335, in PDAC CD44+/CD133+/EpCAM+ (triple-marker-positive) CSCs [184]. In the same study, as a proof of concept, knockdown of miR-125b resulted in the inhibition of tumor aggressiveness, consistent with the downregulation of CD44, EpCAM, EZH2, and snail. These results clearly suggest the importance of miRNAs in the regulation of CSCs characteristics, and their potential role as novel targets to improve therapeutic efficacy.

## 7. Conclusions and Future Perspectives

PDAC is a common cause of cancer-death and has the worst prognosis of any major malignancy, with less than 5% of patients alive 5 years after diagnosis. miRNAs have been documented to be involved in PDAC tumorigenesis; progression and recent evidence support their utility as promising biomarkers in cancer diagnosis and prognosis. In the present review we evaluated studies on the association between candidate miRNAs and drug response/resistance. Importantly, miRNAs remain intact in routinely collected, formalin-fixed, paraffin-embedded tumor tissues, and biofluids, and hopefully, in the near future, the expression profiles of specific miRNAs could provide new information about resistance of individual tumors to different treatments before starting therapy, while modulation of the expression of other miRNAs during treatment might offer a new tool for the prediction of acquired resistance.

However, as with previous studies on gene profiling, most emerging miRNA signatures of chemoresistance are not overlapping and no conclusive evidence has been obtained on their clinical utility. The controversial results might be explained by different specimens (frozen versus paraffin-embedded, micro- versus nonmicrodissected), experimental platforms used (quantitative PCR versus different miRNA array or* in situ* hybridization systems), stage, and regimens as well as small sample size, ethnic differences, and lack of appropriate statistical analyses.

Additional studies in larger homogeneous populations with validated methodology are needed to clarify these issues. Furthermore, new analytical techniques, such as next-generation sequencing, may provide useful tools to understand the role of miRNA as effective biomarkers also starting from very small amount of tissues. The next step will then be to use the emerging miRNAs as markers within prospective trials, to see if they can aid clinical decision-making.

## Figures and Tables

**Table 1:** Selected miRNA candidates which are correlated to gemcitabine chemoresistance in pancreatic cancer.

miRNA	Expression	Targets	Reference
miR-21	Upregulated	EGFR, HER2/neu, PDCD4, BCL2, PTEN, TIMP2, and TIMP3	[139, 142]
miR-222 and miR-221	Upregulated	p27, PUMA, PTEN, and Bim	[84, 185]
miR-10a and miR-10b	Upregulated	HOXB8, HOXA1	[186, 187]
miR-214	Upregulated	PTEN, ING4	[188, 189]
mir-320c	Upregulated	SMARCC1	[190]
miR-155	Upregulated	PI3K SMG-1	[148]
miR-34°	Downregulated	BCL-2	[43]
Let-7	Downregulated	E2F2, c-Myc, KRAS, and MAPK	[125]
miR-142-5p	Downregulated	Unknown	[124]
miR-204	Downregulated	MIC-1	[124]
miR-200a, miR-200b, and miR-200c	Downregulated	EP300	[125, 191]
